# Mathematically optimal decisions in forensic age assessment

**DOI:** 10.1007/s00414-021-02749-y

**Published:** 2021-12-15

**Authors:** Petter Mostad, Andreas Schmeling, Fredrik Tamsen

**Affiliations:** 1 Mathematical Sciences, Chalmers University of Technology, Gothenburg, Sweden; 2 Mathematical Sciences, Gothenburg University, Gothenburg, Sweden; 3 Institute of Legal Medicine, University Hospital Münster, Münster, Germany; 4 Forensic Medicine, Department of Surgical Science, Uppsala University, Uppsala, Sweden

**Keywords:** Age assessment, Third molar, Femur, Knee, Bayesian

## Abstract

Forensic age estimation generally involves considerable amounts of uncertainty. Forensic age indicators such as teeth or skeleton images predict age only approximately, and this is likely to remain true even for future forensic age indicators. Thus, forensic age assessment should aim to make the best possible decisions under uncertainty. In this paper, we apply mathematical theory to make statistically optimal decisions to age assessment. Such an application is fairly straightforward assuming there is a standardized procedure for obtaining age indicator information from individuals, assuming we have data from the application of this procedure to a group of persons with known ages, and assuming the starting point for each individual is a probability distribution describing prior knowledge about the persons age. The main problem is then to obtain such a prior. Our analysis indicates that individual priors rather than a common prior for all persons may be necessary. We suggest that caseworkers, based on individual case information, may select a prior from a menu of priors. We show how information may then be collected over time to gradually increase the robustness of the decision procedure. We also show how replacing individual prior distributions for age with individual prior odds for being above an age limit cannot be recommended as a general method. Our theoretical framework is applied to data where the maturity of the distal femur and the third molar is observed using MRI. As part of this analysis we observe a weak positive conditional correlation between maturity of the two body parts.

## Introduction

Physiological processes, like the growth of bones and teeth, can usually be described in developmental stages that occur in predictable sequences. By studying the prevalence of different stages in subjects of various ages, the stages can be correlated with chronological age. If the studies are large enough and performed on representative populations, such developmental stages can then be used as age indicators in order to predict a person’s chronological age in a medical age assessment [1].

Radiological methods are commonly used to observe age indicators, but there is no consensus between different countries on which techniques to use. In Europe, the most commonly used methods are x-ray of hand, teeth and clavicle [2]. The choice of method is of course a crucial part of an age assessment. If you, for example, want to assess whether a person is above or below the age of 18, it is not helpful to use an age indicator that normally is fully developed at that age. Instead, you need an age indicator that is still under development at whatever age limit you want to assess, so that you can separate those that are below and above that age, respectively.

However important the choice of method is, the statistical analysis of the results as well as the presentation of the conclusions is also fundamental parts of an age assessment. If you choose good techniques but make a poor analysis and presentation, there is a risk that the overall assessment will be inadequate. The combination of radiological methods, statistical analysis, and presentation of the results will be referred to as an age assessment model.

An important age limit in many countries is the age of 18. Whether a person is below or above this age limit often has implications on how an asylum seeker will be handled or a convicted criminal will be sentenced. In addition to different countries using different methods, the way the age indicators are interpreted also differs. In Norway’s model for example, the results from x-ray of the third molar and the hand are combined to get a more precise estimate than by using the two methods on their own [3].

The Study Group on Forensic Age Diagnostics (Arbeitsgemeinschaft für Forensische Altersdiagnostik; AGFAD) recommends the use of three methods: x-ray of hand, x-ray of teeth, and if the hand skeletal development is complete, an additional x-ray or CT of the clavicles. If one needs the highest standard of proof, the concept of minimum age should be applied [4]. This is a conservative way of interpreting the results, giving the benefit of the doubt to younger people.

Given a set of age assessment indicators, there is a need to find an optimal age assessment model, so that an optimal decision procedure can be produced when using these indicators. In many parts of forensic science, a Bayesian paradigm has been adopted, see, e.g., [5]. An example from age assessment is [6]. In this paper we explore some consequences of applying Bayesian decision theory to age assessment in general. We describe a general framework, and conclude that formulation of prior knowledge about the age of each individual is key. We then discuss how this can be done in practice, as part of a practical functioning system for age assessment. One possibility is using a menu of priors, where case workers can select a prior based on the case context. We show how such a system could be sequentially updated with previous cases to become increasingly optimal. On the other hand, we also show how using prior odds instead of prior distributions for ages will result in clear suboptimalities.

We illustrate our computations using a dataset where the maturity of the distal femur and the third molar is observed using MRI. Some study subjects or cohorts have been previously reported in [7–9].

## Materials and methods

### Data

A total of 542 German male and female volunteers aged 12 to 24 years were prospectively examined from May 2013 to March 2015 at the Department for Clinical Radiology of the University Hospital of Münster, Germany. The study received a positive vote by the relevant ethics committee (reference number: 2013-062-f-S). After being duly informed, all study participants gave their written consent to take part in the study. For minors, the written consent of the parents was also obtained.

Examinations were performed on a Philips 3.0 T Achieva (gradient amplitude 80 mT/m, Philips Medical Systems, Netherlands). With regard to the knee examination, the primary region of interest was the left knee joint with the possibility of switching to the right knee joint in case of unilateral exclusion criteria (i.e., trauma or implant). The teeth MRI examination was primarily made on the third molar of the left lower quadrant. For study participants whose medical case history indicated that their left lower third molar had been extracted, the third molar of the right lower quadrant was examined.

Knee images were acquired with a T1-weighted turbo spin echo (TSE) sequence in coronal orientation (TR 633 ms; TE 20 ms; flip angle 90; duration 3:51 min; measured voxel size 0.6 × 0.77 × 3 mm; reconstructed voxel size 0.31 × 0.31 × 3 mm). The development stages were classified according to Schmeling et al. [10] with the addition of the substages according to Kellinghaus et al. [11].

Teeth images were acquired with the high-resolution surface coil SENSE-NV 16 and MRI scans were performed utilizing a T2 turbo spin echo (TSE) sequence (TSE factor = 13; TR = 2800 ms; TE = 80; flip angle = 90; sense = 1.5; NSA = 6; scan duration = 5:36 min; measured voxel size = 0.50 × 0.65 × 2.00 mm; reconstructed voxel size = 0.19 × 0.19 × 2.00 mm). The mineralization stages of the third molars were assessed according to Demirjian et al. [12].

Knee images were assessed by an examiner with experience in musculoskeletal MRI diagnostics and teeth images were assessed by a dentist experienced in third molar mineralization assessment. For both knee and teeth images, subsets were used to assess inter- and intraobserver agreement. An overview of the data is given in Table 1. For more details, see [7] and [8].

**Table 1:** The number of individuals with each type of observation in our dataset

Females	A	B	C	D	E	F	G	H	Sum
2c	0	0	6	11	10	2	0	0	29
3a	0	0	4	8	5	4	0	0	21
3b	0	0	1	1	6	1	0	0	9
3c	0	0	4	3	8	5	0	1	21
4	0	0	1	9	20	40	85	28	183
Sum	0	0	16	32	49	52	85	29	263
Males									
2c	1	4	17	26	20	6	0	0	74
3a	0	0	1	6	8	9	2	0	26
3b	0	0	0	0	0	0	0	1	1
3c	0	0	0	0	4	13	7	2	26
4	0	0	0	0	4	13	72	63	152
Sum	1	4	18	32	36	41	81	66	279

The columns indicate the molar maturity levels A–H and the rows indicate the knee maturity levels observed in these data: 2c–4

### Decision theory for forensic age assessment

We will apply mathematical decision theory to the problem of making optimal decisions about the age of a person when the decision is based on a forensic age assessment report, while all other information about the age is collected into an individual prior represented as a probability density *p*(*x*) on the true age *x*. We will assume there is a finite list $r_{1},r_{2},\dots ,r_{K}$ of possible age assessment reports. Thus, given *p*(*x*), a decision rule assigns to each of these reports a decision of whether the person is above some age limit; in this paper 18 years.

The probability of obtaining report *r*_*k*_ will of course depend on the age *x* of a person, but it may also depend on a number of other covariates. If these covariates are easily observable, such as gender, one should include them in the model. For any true age *x* and covariate *z*, and for $k=1,\dots ,K$, we assume there is a probability *f*_*k*_(*x*,*z*) that the report will be *r*_*k*_. Thus, we have ${\sum }_{k=1}^{K}f_{k}(x,z)=1$ for all fixed *x* and *z*. In “?? ??,” we look at how we can estimate such functions from data.

To differentiate between good and bad decisions, we introduce *benefits* and *expenses* related to correct and incorrect decisions. Let *b*(*x*) be the benefit of making the correct decision for a person of age *x*, and let *e*(*x*) denote the expense of making the wrong decision. Thus, for example, if *x* < 18, *e*(*x*) is the expense of deciding that a person of age *x* is above 18, while *b*(*x*) is the benefit of deciding that the person is below 18. We can now formulate the expected benefits of a classification: For report *r*_*k*_, if we classify as a child, the expected net benefit is

$$ {\int}_{0}^{18}p(x)f_{k}(x,z)b(x) dx - {\int}_{18}^{\infty} p(x)f_{k}(x,z)e(x) dx $$

while if we classify as an adult, the expected net benefit is

$$ {\int}_{18}^{\infty} p(x)f_{k}(x,z)b(x) dx - {\int}_{0}^{18} p(x)f_{k}(x,z)e(x) dx. $$

We should classify as an adult if and only if the expected net benefit of classifying as an adult is greater than the expected net benefit of classifying as a child, i.e., if

$$ \begin{array}{@{}rcl@{}} && {\int}_{18}^{\infty} p(x)f_{k}(x,z)(b(x)+e(x)) dx \\ &&\qquad >{\int}_{0}^{18} p(x)f_{k}(x,z)(b(x)+e(x)) dx. \end{array} $$

We see that we can subsume the benefits of making a correct decision into the expenses of making an incorrect decision. Defining an overall cost function for misclassification *c*(*x*) = *b*(*x*) + *e*(*x*) we get that the condition for classifying as adult becomes

$$ {\int}_{18}^{\infty} p(x)f_{k}(x,z)c(x) dx >{\int}_{0}^{18} p(x)f_{k}(x,z)c(x) dx. $$

The cost function may depend on *x* in any way, but its most important feature is generally to compare the cost of classifying a child as adult to the cost of classifying an adult as a child. Let us write

$$ c(x) = \left\{\begin{array}{ll}Bc_{0}(x) & \text{if $x<18$} \\ c_{0}(x) & \text{if $x\geq18$} \end{array}\right. $$

where *B* is a constant. If *c*_0_(*x*) is symmetric around *x* = 18 then *B* represents the quotient of the cost of classifying a child as an adult divided by the cost of classifying an adult as a child. We get that we should classify as an adult if and only if

1 $$ \frac{{\int}_{18}^{\infty} p(x)f_{k}(x,z)c_{0}(x) dx}{{\int}_{0}^{18} p(x)f_{k}(x,z)c_{0}(x) dx} > B. $$

### The value of *B* and the cost function

To use Eq. 1 a value for *B* must be established. As mentioned, the value of *B* should represent the “cost” of classifying a child as an adult divided by the “cost” of classifying an adult as a child. Here, “cost” refers to all kinds of costs, both for the individual and for society, and including both monetary and ethical “costs.” Of course, the latter can be very difficult to put a number on.

In several guidelines [2, 4] concerning age assessment it is explicitly stated that it is more important to avoid errors where children are assessed as adults than to avoid errors where adults are assessed as children. This corresponds to *B* being above 1. In criminal justice, the principle is often stated that it is more important to avoid convicting innocents than to convict those that are guilty. According to the eighteenth century English jurist William Blackstone, “It is better that ten guilty persons escape than that one innocent suffer.” [13]. Transferring this to our context, it would correspond to a *B* value of 10 or more. Fundamentally, choosing a number *B* is an ethical decision, well beyond the scope of this paper. We will simply assume that reasonable values for *B* lie between 1 and 10, and use this to guide our illustrations.

In most of what follows, we will assume that *c*_0_(*x*) does not depend on *x*. In “Robustness of results,” we look at some consequences of another choice for this function.

### Models for age assessment report probabilities

We now describe a method for obtaining from data functions *f*_*k*_(*x*,*z*) representing the probability for report *r*_*k*_ for a person of age *x* and gender *z*. Our example data contains measurements of knee and teeth maturity for each individual. However, the formulas below can fairly easily be adapted to situations where a different pair of age indicators is observed, or indeed to situations where any number of age indicators are used.

To motivate our model, we imagine that, in addition to chronological age, any person also has a *knee age* and a *tooth age*. Knee age is modelled as chronological age plus a person-specific random variable with expectation zero, and should reflect how well developed the knee is for the person relative to their age. The knee age is not directly observed, instead, one observes if it has passed certain threshold values *a*_1_,*a*_2_,*a*_3_,*a*_4_. Specifically, if the knee age is below *a*_1_ one observes the knee development stage 2c. If it is between *a*_1_ and *a*_2_, one observes stage 3*a*, etc. Covariation between the development of knees and teeth is modelled as a covariance between the random variables used to obtain the knee and tooth ages from the chronological age.

Let us first consider only the knee age, and let us use a model where the random variable is normally distributed with a fixed variance ${\sigma ^{2}_{k}}$. Mathematically, if the pair (*x*,*y*_1_) represents the chronological age and the knee age indicator for a person (coded as an integer 0 ≤ *j* ≤ 4), we model

$$ y_{1} = {\max}_{j\in\{0,\dots,4\}}\left\{j:a_{j}< x + u_{1}\right\} $$

where $u_{1}\sim \operatorname {Normal}(0,{\sigma ^{2}_{k}})$ and we define $a_{0}=-\infty $. Using flat priors on $a_{1},\dots ,a_{4}$ and the standard improper prior for ${\sigma ^{2}_{k}}$ that is proportional to $1/{\sigma ^{2}_{k}}$ we can use a sample of data values (*x*,*y*_1_) and, for example, Gibbs sampling to obtain posterior values for the model parameters.

To understand results from such a method, one may consider Figs. 2 and 3, which illustrate results when the method is applied to the females of our data set and the observations of their knee maturity. In Fig. 3, each curve shows the probability at a given chronological age of observing a specific age indicator (for example, stage 3c) *or something less mature*. In Fig. 2 each curve shows the probability of observing each specific age indicator. Note how the age at which the curves in Fig. 3 pass the value 0.5 correspond to the cutoff values $a_{1},\dots ,a_{4}$. The model is identical to the one used in [14] except that the model above does not include the possibility of missing data. The model is also similar to other models used in the literature where the populations of persons having a maturity stage are modelled as normally distributed. Note how the formulation above solves the problem of observations for the initial and final stages not being normally distributed.

When both a knee indicator *y*_1_ and a tooth indicator *y*_2_ is observed for each person (with the tooth maturity levels A through H represented as integers 0 through 7) we use the model

2 $$ \begin{array}{@{}rcl@{}} y_{1} &=& {\max}_{j\in\{0,\dots,4\}}\left\{j:a_{j}<x + u_{1}\right\} \end{array} $$

3 $$ \begin{array}{@{}rcl@{}} y_{2} &=& {\max}_{j\in\{0,\dots,7\}}\left\{j:b_{j}<x + u_{2}\right\} \end{array} $$

where $(u_{1},u_{2})\sim \operatorname {Normal}_{2}(0, {\Sigma })$ so that the pair has a bivariate normal distribution, and use a standard improper prior proportional to $\lvert {\Sigma } \rvert ^{-1}$ on the covariance matrix Σ.

### Individual age priors

The remaining part of Eq. 1 to be discussed is the individual age prior *p*(*x*). The purpose of establishing individual priors is to be able to mathematically combine the information from a forensic age assessment with some of the other individual information regarding age that in practice is available in every case. Guidelines [2] for forensic age determination generally stress that all persons have the right to an individual assessment. If medical age assessment data always had strong evidential weight, the individual assessment could be limited to such data. Unfortunately, the evidential weight from medical age assessment data is often limited. Indeed, as we will see in “?? ??,” the optimal decision may often differ when switching from one reasonable age prior to another. In other words, in order to make good and fair decisions, we need to find practical ways to represent the “other information” mentioned above in terms of age priors.

Below, we discuss two options. First, we discuss how case workers might use case data to select an age prior from a menu of priors. We also discuss using prior odds for the age being above or below the cutoff.

#### A menu of priors

We propose to divide individuals into groups based on why a forensic age assessment is performed for them, and circumstances in their background. For example, for refugees, the groups may be based on the country of origin. Differences in priors chosen for different groups may then be motivated by previous experience with age assessment of persons in this group. In “Using priors from a list of priors,” we exemplify how one might use 12 different priors for 12 different groups.

The prior may if necessary be adjusted based on individual circumstances. For example, there may be documented observations of medical, psycho-social, or other types of maturity not conforming to the standards used in the age assessment reports. Although challenging to do in a fair and structured manner, such information could result in specific changes to the individual prior. There might also be case circumstances leading to well documented upper or lower bounds on the age, which could then be included in the prior.

#### Using experience to improve decisions

Assigning different priors to persons in different groups must somehow be done based on differences in experiences with these groups. Indeed, as more persons are assessed, accumulated information may be used to improve the priors in a precise manner, as explained below.

Assume *N* individuals in a group with fixed covariate *z* have been assessed, resulting in reports $R_{1},\dots ,R_{N}$, respectively. Let $q_{1},\dots ,q_{Q}$ be a set of reasonable possible priors for the group. We would like to estimate from the reports positive weights $\gamma _{1},\dots ,\gamma _{Q}$, summing to 1, so that ${\sum }_{j=1}^{Q}\gamma _{j}q_{j}(x)$ is a good prior for the group. In fact, if such a weighted sum was the true age distribution, the data would be multinomially distributed with probability

4 $$ \begin{array}{@{}rcl@{}} p_{k} &=& {\int}_{0}^{\infty} f_{k}(x,z){\sum}_{j=1}^{Q}\gamma_{j} q_{j}(x) dx\\ &=& {\sum}_{j=1}^{Q}\gamma_{j}{\int}_{0}^{\infty} f_{k}(x,z)q_{j}(x) dx \end{array} $$

for each of the *K* possible reports. Thus, the observed data yields a likelihood on the set of possible *γ* vectors. Using a flat prior, we may compute, for example, the expected posterior value for *γ*, using, for example, MCMC simulation.

In “Using prior odds instead of prior densities,” we explore the practicality of this approach in a simulation study: For a hypothetical group of persons with true age distribution *π*(*x*), we simulate the ages of *N* persons. Using the functions *f*_*k*_(*x*,*z*) for males from “?? ??” to simulate observations for each individual, we get a set of *N* simulated reports. We then use the procedure above, letting $q_{1},\dots ,q_{Q}$ be the priors of Table 3. This leads to a specific mixture, which we can then compare with the original true age distribution *π*(*x*).

#### Using prior odds

Above, we have discussed how to obtain individual prior distributions *p*(*x*). Some disadvantages of the approach of “A menu of priors” are that a rather ad-hoc list of priors has to be assembled by somebody, and that the connection between case information and these probability densities may not be transparent for case workers.

A slightly easier concept than prior probability densities for ages is the concept of prior odds. One then considers the prior probability that the person is above 18 divided by the prior probability that the person is below 18. As this is a single number, it may be easier for case workers to understand and develop experience with. We discuss below how to make optimal decisions if prior odds are selected for each individual, while other features of the prior distribution *p*(*x*) are standardized to be equal for all individuals.

For any prior *p*(*x*) we can write

$$ p(x) = \Pr(x\!<\!18)p(x\mid x\!<\!18) + \Pr(x\!\geq\!18)p(x\mid x\!\geq\!18) $$

where, for example, $\Pr (x<18)$ is the probability that the age is less than 18 and *p*(*x*∣*x* < 18) is the conditional probability density of ages given that the age is less than 18. Replacing *p*(*x*) with this expression in Eq. 1 we get that we should classify as adult if and only if

5 $$ \frac{\Pr(x\geq18)}{\Pr(x<18)} \cdot \frac{{\int}_{18}^{\infty} f_{k}(x,z)p(x\mid x\geq18)c_{0}(x) dx} {{\int}_{0}^{18}f_{k}(x,z)p(x\mid x<18)c_{0}(x) dx}>B.  $$

Equation 5 can be interpreted as follows: The left-hand side represents the strength of the total evidence for adulthood. To make the decision for adulthood, this value needs to exceed *B*, which represents how serious we look at misclassifications of children as adults compared to misclassifications of adults as children. The strength of the total evidence for adulthood is separated into the prior odds of adulthood, represented by the first factor, and a factor that is connected to the evidence from the forensic assessment. If the prior odds for adulthood is high, less strong evidence from the forensic assessment is needed to reach the threshold *B* in order to make the decision for adulthood, and vice versa.

The second term of Eq. 5 also depends on *p*(*x*), in the form of the conditional densities *p*(*x*∣*x* ≥ 18) and *p*(*x*∣*x* < 18). Is this dependency in practice small or large? In other words, if we make the approximation that we use the same standard conditional densities *p*(*x*∣*x* ≥ 18) and *p*(*x*∣*x* < 18) for all persons, while using individual prior odds, will this approximation influence optimal decisions to an acceptably small extent? This is the question we explore in “Using prior odds instead of prior densities.”

## Results

Results are divided into five sections. In “?? ??,” we present the results when applying the methods of “?? ??” to our dataset, including the estimation of the functions *f*_*k*_(*x*,*z*) relating chronological age *x* and gender *z* to the probability of obtaining a report type *r*_*k*_. Some interesting features of these results are that they contain information about the correlation between tooth and knee development, and about consequences of using MRI for tooth maturity assessment.

In “Using priors from a list of priors,” we exemplify results when using a menu of age priors as suggested in “?? ??,” and indicate how such a menu can be used in practical decision making. In “?? ??,” we use the ideas of “?? ??” to exemplify how a system using a menu of age priors can be robustified with use.

“Using prior odds instead of prior densities” presents results when using the idea from “Using prior odds” with individual prior odds instead of individual priors. The final “Robustness of results” looks at the robustness of our conclusions relative to some of the assumptions we have used.

### The probability of obtaining a report as a function of chronological age

The model of “?? ??” was applied separately to the male and female data presented in “Data.” The first two tables in Table 2 list the expected values for $a_{1},\dots ,a_{4}$ and $b_{1},\dots ,b_{7}$ in our model, corresponding to the ages at which 50% of persons reach a given maturity level. We also list 95% credibility intervals. Note that these are the uncertainty intervals for the threshold values due to limited amounts of data.

**Table 2:** The age at which 50% from the listed gender reaches the listed maturity level for the listed age indicator

	Knee stage				Tooth stage								
	3a	3b	3c	4	B	C	D	E	F	G	H	Knee transitions	Tooth transitions
Males	15.4	16.8	16.9	18.3	8.0	10.1	12.3	14.3	16.4	18.6	22.2	1.5	2.1
95% interval	15.0–15.8	16.4–17.2	16.5–17.3	18.0–18.7	5.7–9.7	8.9–11.0	11.6–12.9	13.8–14.8	15.9–16.8	18.1–19.0	21.8–22.7	1.3–1.7	1.8–2.4
Females	14.0	15.2	15.8	16.9	–	–	12.1	14.7	17.3	19.9	24.3	1.0	2.6
95% interval	13.7–14.4	14.9–15.5	15.4–16.1	16.5–17.2	–	–	11.1–12.9	14.0–15.3	16.8–17.8	19.4–20.4	23.6–25.0	0.8–1.2	2.2–3.0

The intervals given below the numbers are 95% credibility intervals reflecting the uncertainty due to limited amounts of data. The third table lists the time interval covering 95% of all transitions from one maturity stage to the next. Note that no females in the data have teeth stages A or B

Regarding the final stages, i.e., knee stage 4 and tooth stage H, the median ages of attainment have previously been estimated in other studies, see, e.g., [14]. For the knee, an age of 18.5 years was estimated from a study conducted by the Swedish National Board of Health and Welfare [15]. This is similar to the age found in the present data set. The tooth stage of H, however, is attained at a significantly higher age in the present study compared to previous ones [16–18]. An important difference between these studies and the present one is that they used conventional x-ray while our data comes from MRI examinations. A previous study comparing dental staging with x-ray and MRI, respectively, did not find any statistically significant difference between the methods [19]. Therefore, the results found in our data are somewhat surprising.

The observed discrepancy between the results in Table 2 and the results in other studies is not in itself a problem for age assessment, as long as one follows the procedure recommended in this paper, where assessment decisions are based on data obtained with exactly the same measurement procedure as the one used on those for which decisions shall be made. But it underscores the danger of using decision procedures based on results from studies that use different observational procedures of the same age indicator.

Our model also gives us posterior information about the variation of “tooth age” and “knee age” relative to chronological age, using the concepts from “?? ??.” For example, considering knees of males, there is for each male a time point when the knee development goes from one stage to the next. Of course this time point varies between males, and our model indicates that a time interval of length 1.5 years covers 95% of all such transition time points. The last part of Table 2 lists the results for both genders and both indicator types, together with the uncertainty in the results due to limited data. Note that it is an assumption of our model that the lengths of these intervals are the same for all transitions for a given gender and body part.

Finally, our model gives an indication of the correlation between the difference between “tooth age” and chronological age, and the difference between “knee age” and the chronological age. This can be interpreted as the correlation between tooth and knee development. Note that we are here talking about the correlation conditional on age: Obviously, observing a later knee stage for a person increases the probability of observing a later tooth stage for that person, and vice versa. In contrast, conditional correlation addresses the following question: For persons at a specific chronological age, will those who have a late knee stage for their age also tend to have a late tooth stage for their age?

**Fig. 1:**
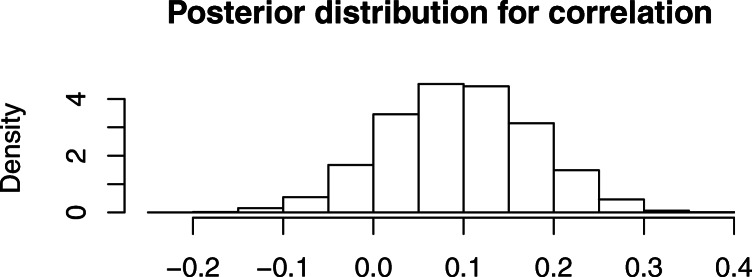
The inferred conditional correlation between knee and tooth values for males

The expected posterior conditional correlation between tooth age and knee age for males is 0.1, with a 95% credibility interval (− 0.06,0.25), so that there is an 88% posterior probability that the correlation is positive. Figure 1 shows the posterior distribution of the correlation. This result is not strong enough to prove that there is a positive conditional correlation, but it points in that direction. However, the correlation seems to be weak, so using models without conditional correlation may be acceptable as an approximation.

**Fig. 2:**
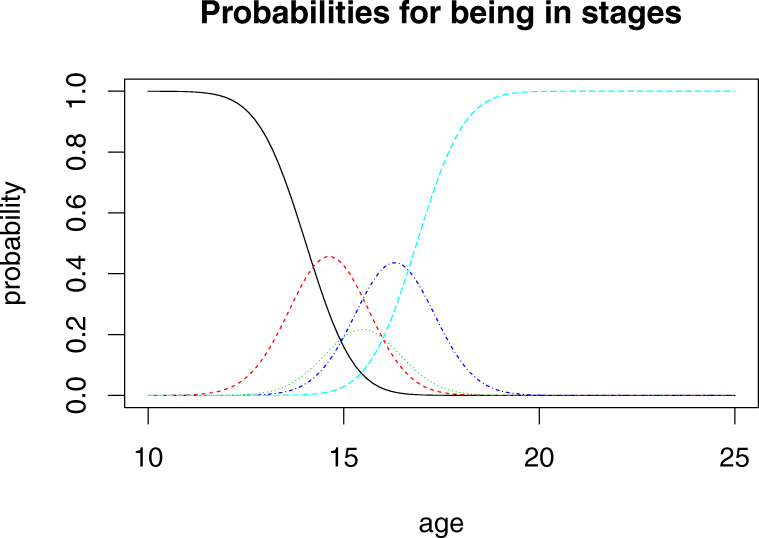
Female probabilities for being in knee stage 2c, 3a, 3b, 3c, 4 as a function of chronological age

**Fig. 3:**
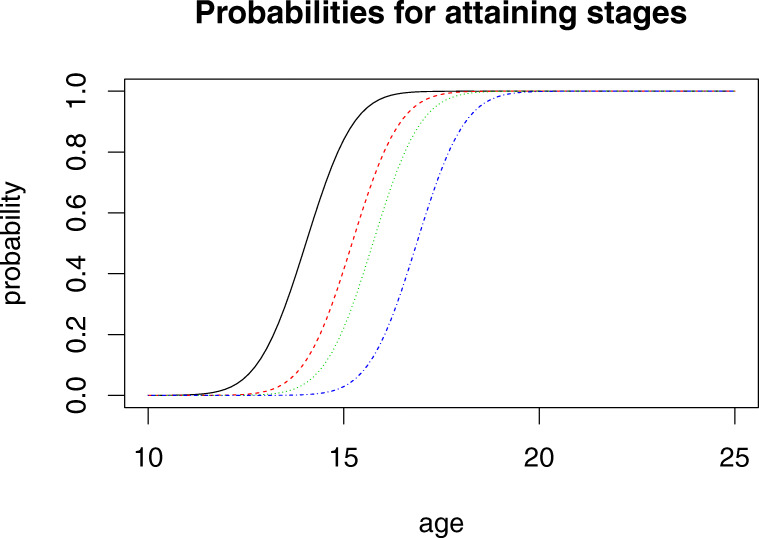
Female probabilities for reaching knee stage 3a, 3b, 3c, 4 as a function of chronological age

In this paper, the main purpose of the data analysis is to derive functions *f*_*k*_(*x*,*z*) which can be part of an age assessment procedure. As we use 5 possible knee stages and 8 possible tooth stages, we have a set of 40 possible reports. For each such report, and for each gender, our model produces a function of *x* which for each age *x* gives the probability that a person of the given gender has the corresponding combination of maturity stages. Figure 2 illustrates what such curves look like. Here, we have summed over the probabilities for the tooth stages and only focus on the knee stages. The results may also be illustrated with curves indicating cumulative probabilities, so in this case, the probability for females to have specific knee maturity stages or something less. Figure 3 shows such results corresponding to those of Fig. 2.

### Using priors from a list of priors

Choosing specific priors that correspond to specified groups of persons to be age assessed is a project beyond the scope of this paper. Instead, we will simply use a rather ad-hoc list of priors to illustrate the computational consequences of using such a menu of priors. Specifically, the priors listed in Table 3 are used. For each of these priors and for each of a selection of 9 likely reports, the value of the left side of Eq. 1 has been computed for males and listed in Table 4.

**Table 3:** The example priors used

Function	Distribution	Prior prob. for adult	Mean	Standard deviation
*p*_1_(*x*)	Triangle(15, 18, 25)	0.7	19.33	2.09
*p*_2_(*x*)	Normal(19.05, 2)	0.7	19.05	2
*p*_3_(*x*)	Normal(18.52, 1)	0.7	18.52	1
*p*_4_(*x*)	Triangle(13.92, 16.92, 23.92)	0.5	18.25	2.09
*p*_5_(*x*)	Normal(18, 2)	0.5	18	2
*p*_6_(*x*)	Normal(18, 1)	0.5	18	1
*p*_7_(*x*)	Triangle(12.58, 15.58, 22.58)	0.3	16.91	2.09
*p*_8_(*x*)	Normal(16.95, 2)	0.3	16.95	2
*p*_9_(*x*)	Normal(17.48, 1)	0.3	17.48	1
*p*_10_(*x*)	Triangle(16.27, 19.27, 26.27)	0.9	20.60	2.09
*p*_11_(*x*)	Normal(20.56, 2)	0.9	20.56	2
*p*_12_(*x*)	Normal(19.28, 1)	0.9	19.28	1

**Fig. 4:**
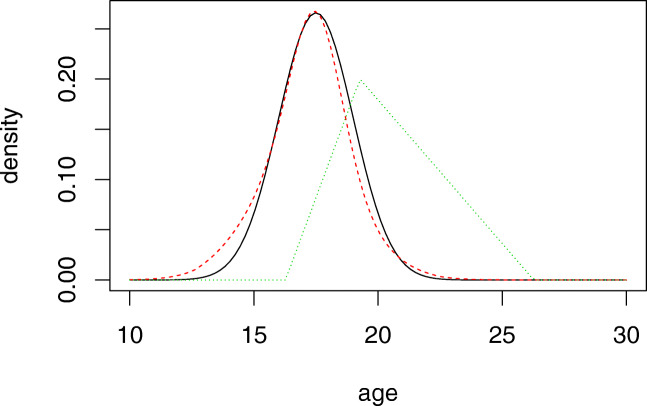
Various prior age distributions. The full line indicates the true age distribution used in the simulation experiment. Initially, the dotted green triangular line is used as a prior; it is the prior *p*_10_ from the list of Table 3. Based on the information of 1000 simulated cases in this group, the red dotted prior is obtained, following closely the true prior

The three first have been chosen to have variable form and variance, while still having a 0.7 prior probability for adulthood. All the remaining priors are translations of the first three, where the translations have been chosen so that the prior probability for adulthood is 0.5, 0.3, and 0.9, respectively. The density *p*_10_(*x*) illustrated in Fig. 4

**Table 4:** The table shows the left-hand side of Eq. 1 using the listed priors (see Table 3) and the listed forensic report results for a male

	(3c, E)	(3c, F)	(4, E)	(3c, G)	(4, F)	(3b, H)	(4, G)	(3c, H)	(4, H)
*p*_1_(*x*)	0.09	0.15	0.17	0.25	0.27	0.45	0.47	0.87	1.74
*p*_2_(*x*)	0.08	0.15	0.17	0.27	0.30	0.51	0.53	1.03	2.13
*p*_3_(*x*)	0.26	0.37	0.41	0.56	0.59	0.88	0.90	1.44	2.41
*p*_4_(*x*)	0.03	0.06	0.08	0.12	0.14	0.24	0.26	0.50	1.08
*p*_5_(*x*)	0.04	0.08	0.10	0.16	0.18	0.30	0.33	0.63	1.28
*p*_6_(*x*)	0.12	0.18	0.20	0.28	0.30	0.44	0.46	0.73	1.20
*p*_7_(*x*)	0.02	0.04	0.04	0.07	0.09	0.15	0.18	0.36	0.82
*p*_8_(*x*)	0.02	0.05	0.05	0.09	0.10	0.18	0.20	0.38	0.78
*p*_9_(*x*)	0.05	0.08	0.09	0.13	0.14	0.21	0.23	0.36	0.60
*p*_10_(*x*)	0.33	0.47	0.54	0.73	0.76	1.25	1.21	2.23	4.31
*p*_11_(*x*)	0.18	0.31	0.36	0.55	0.59	1.05	1.04	2.13	4.62
*p*_12_(*x*)	0.71	0.99	1.11	1.50	1.55	2.45	2.39	4.13	7.32

The optimal classification is “adult” whenever the number is above the chosen value for *B*

To use Table 4 in practical work, the person to be assessed needs to be put in one of the groups corresponding to one of the priors. A number can then be read from the table based on the result of the forensic report for the person. If this number is above the chosen value for *B*, the optimal decision is to classify as an adult; otherwise, one should classify as a child.

The first thing to notice from the table is that most values are fairly low, and none are above 10. This shows that a careful ethical consideration of the proper value of *B* is unavoidable, when the forensic data is of this type. For example, if one sets *B* = 10, to be “on the safe side” and to avoid children being classified as adults, the conclusion would be that all assessed persons should be classified as children.

Secondly, we notice that the choice of prior generally matters when deciding what the optimal conclusion is. The values in each column generally vary by an order of magnitude. For many report results the corresponding column contains values both below and above reasonable *B* values. Thus, the process of selecting a prior for each individual seems unavoidable. As mentioned, the process may be facilitated by specifying groups of persons and connecting a prior to each group. A person to be assessed then needs to be placed in one such group.

We may also notice that numbers generally increase from left to right in the table. Looking at the table heading, we see that reports indicating more mature body parts generally give higher numbers, as expected. However, the ordering of the reports is not perfect, and may be influenced by the choice of prior. For example, using most priors, the combination (4,*G*) can be considered more mature than the combination (3*b*,*H*). However, for the priors *p*_10_, *p*_11_, and *p*_12_, this ordering is reversed.

### Using a menu of priors together with previous results

We saw in the previous section that the choice of age prior for a person is of profound importance for the result. Thus, it is of high importance to create a practical and fair way to assign individual priors. We have proposed to create groups of persons and to assign a prior to each such group. We now show in a simulation experiment that even if the initial assignment of a prior is imperfect, one can use assessment results from the group to improve decisions over time.

Assume a specific group of males have a true age density that is normally distributed with expectation 17.5 and standard deviation 1.5. This distribution is illustrated with the full line in Fig. 4. Initially, the prior *p*_10_ from Table 3 is used for the group. We see in Fig. 4 that the choice of this prior is not a good one. Indeed, in Table 5 you can compare the values for the left-hand side of Eq. 1 computed with the true prior and the *p*_10_ prior; they may very well result in different decisions in some cases.

**Table 5:** The table shows the left-hand side of Eq. 1 using the true age distribution in the simulation experiment, the prior *p*_10_ used for initial computations, and the prior learned from 1000 simulated cases in the group

	(3c, E)	(3c, F)	(4, E)	(3c, G)	(4, F)	(3b, H)	(4, G)	(3c, H)	(4, H)
True	0.04	0.07	0.08	0.13	0.14	0.23	0.26	0.45	0.86
Initial	0.33	0.47	0.54	0.73	0.76	1.25	1.21	2.23	4.31
Learned	0.03	0.06	0.07	0.11	0.12	0.20	0.22	0.39	0.73

The ages of 1000 persons are then simulated, using the true age distribution, and forensic results are simulated for each based on their ages. Using this data and the methods of “Using experience to improve decisions,” we obtained the weighted prior illustrated with the red dotted line in Fig. 4. We see it follows the true prior much more closely. Indeed, the values computed with this prior in Table 5 are much closer to the correct values obtained with the true age distribution.

### Using prior odds instead of prior densities

In “Using prior odds,” we discussed the idea of using individual prior odds instead of using individual prior distributions. Such a simplification has several practical advantages. Computations for each person becomes simpler, and it should be easier for case workers to understand and use the concept of a prior probability for adulthood rather than using a menu of prior densities.

However, it is clear from Table 4 that the simplification cannot be recommended. As we can see from Table 3, the 12 priors are organized into 4 groups of three, with the priors in each group having the same prior odds (i.e., the same prior probability for adulthood). The suggested simplification would imply that the conclusions for the three priors and for a given report would be the same. However, in Table 4, we see that these three numbers in many cases are quite different, and may be on either side of a reasonable threshold for *B*. So a simplification using a method based on prior odds instead of prior densities would necessarily be suboptimal in a considerable number of cases.

### Robustness of results

So far, we have assumed that the cost function *c*_0_(*x*) is constant. However, the “cost” of mis-classifying somebody who is 17.9 years may be perceived as smaller than the “cost” of mis-classifying somebody who is 16. This may be represented by a varying cost function *c*_0_(*x*): If the cost is only 10% then one should have *c*_0_(17.9) = 0.1 ⋅ *c*_0_(16). Various models may be considered for *c*_0_(*x*). Table 6 shows the version of Table 4 where we have used the cost function

6 $$ c_{0}(x) = |18-x|  $$

Generally, the numbers seem to get more extreme, i.e., small numbers get smaller and large numbers get larger. However, the main features of the two tables are very similar.

**Table 6:** The table is similar to Table 4, but now the cost function of Eq. 6 is used

	(3c, E)	(3c, F)	(4, E)	(3c, G)	(4, F)	(3b, H)	(4, G)	(3c, H)	(4, H)
*p*_1_(*x*)	0.03	0.06	0.07	0.12	0.14	0.31	0.32	0.88	2.64
*p*_2_(*x*)	0.02	0.05	0.07	0.13	0.15	0.34	0.36	1.04	3.23
*p*_3_(*x*)	0.12	0.20	0.24	0.38	0.41	0.78	0.79	1.69	3.72
*p*_4_(*x*)	0.01	0.02	0.02	0.05	0.05	0.12	0.13	0.38	1.25
*p*_5_(*x*)	0.01	0.02	0.03	0.06	0.07	0.16	0.17	0.48	1.46
*p*_6_(*x*)	0.04	0.07	0.08	0.13	0.14	0.27	0.28	0.58	1.26
*p*_7_(*x*)	0.00	0.01	0.01	0.02	0.03	0.06	0.07	0.21	0.73
*p*_8_(*x*)	0.00	0.01	0.01	0.03	0.03	0.07	0.08	0.22	0.67
*p*_9_(*x*)	0.01	0.02	0.03	0.04	0.05	0.09	0.10	0.20	0.43
*p*_10_(*x*)	0.23	0.38	0.46	0.74	0.78	1.71	1.57	4.11	11.01
*p*_11_(*x*)	0.07	0.16	0.20	0.38	0.42	1.07	1.02	3.24	10.53
*p*_12_(*x*)	0.55	0.93	1.11	1.77	1.87	3.83	3.65	8.44	19.52

## Practical application and discussion

A medical age assessment can be reported in different ways, for example, as the minimum age, the most probable age, or as the probability of the person being below or above some age limit. The last alternative may seem attractive when the question is whether a person is a child or an adult. This approach does however require that an assumption is made about the age distribution of the population from which the person comes, e.g., a population of asylum seekers. The requirement may seem counter-intuitive, since the reason why an age assessment is performed on a population is that their ages are unknown. Nevertheless, it is not meaningful to speak about probabilities of being below or above an age limit without such an assumption.

A previous study has shown that it is possible to draw conclusions about the age distribution in a population with unknown chronological ages, given the distribution of their age indicators in previously performed medical age assessments [14]. In the present study, it is shown that given a set of age indicators, the choice of prior age distribution may significantly influence the probability that the person is below or above the age of 18 years. Thus, the prior distribution must be chosen with care.

In this context, it is important to remember that an age assessment should include all available information, not only the medical part. One way to take non-medical information into account could be to let it influence the choice of the prior age distribution. Ideally, an individual prior should be chosen based on the known information about a specific individual. However, such a solution may not be practically feasible due to the difficulty of the task and the time it would take.

Another possibility would be to have a set of priors from which one is chosen, depending on the non-medical information in the specific case. If there, for example, is relatively strong documentation supporting a minor age together with supporting testimonial from teachers, a prior in which the majority of individuals are under 18 years could be chosen. Conversely, if there is no documentation supporting an age below 18 together with testimonials indicating an age above 18, a prior in which a majority are adults could be chosen. If the non-medical information does not point in any direction, a population with half children, half adults might be the best choice.

The concept of prior distributions and their effect on the probability assessment may be hard for people to understand. Nevertheless, if such probabilities are used, this concept is unavoidable. If there are indications that the recipients of the medical age assessments, for example, the migration agency or the courts, cannot evaluate them in a correct way, it is perhaps better to use other ways of communicating the assessments. The minimum age concept is, for example, easier to understand, but conservative.

Decision makers may benefit from considering results that start from chronological age and look at the probability of having different age marker stages at that age. If a combination of age markers is used, this can be presented as the proportion of individuals in each age group (e.g., 17-year-olds) that have each combination of age markers (for example, knee stage 4 and third molar stage H). This way of presenting the results may give recipients that are not well-versed in statistics a better understanding of the precision of the methods.

## Conclusions

Given a set of age indicator and an assumption of prior age distribution, we have created a theoretical framework to make statistically optimal decisions in medical age assessment. A prior distribution is inherently difficult to assume since the people being assessed have unknown ages. However, speaking about probabilities of a person being below or above an age limit without such an assumption is meaningless. We have shown that the choice of the prior age distribution may significantly affect the medical age assessment. Making sound and transparent assumptions about priors is therefore of great importance to the rule of law, if such probabilities are to be used.

Regarding the data set we use, our results are consistent with some small positive conditional correlation between knee maturity and tooth maturity, but no firm conclusions regarding this can be drawn from our analysis. We see that using the present MRI technique for observation of tooth maturity tends to result in classifications where maturity occurs at a later age, compared to some previous studies using x-ray observations.
